# Neoadjuvant chemotherapy for carcinoma of the oesophagus and oesophago-gastric junction: a six-year experience

**DOI:** 10.1186/1477-7800-4-24

**Published:** 2007-10-16

**Authors:** Brian P Halliday, Richard JE Skipworth, Lucy Wall, Hamish A Phillips, Graeme W Couper, Andrew C de Beaux, Simon Paterson-Brown

**Affiliations:** 1 Department of Surgery, Royal Infirmary of Edinburgh, 51 Little France Crescent, Edinburgh, EH16 4SA, UK; 2 Department of Oncology, Western General Hospital, Crewe Road, Edinburgh, EH4 2XU, UK; 3 Consultant Surgeon and Honorary Senior Lecturer, Department of Surgery, Royal Infirmary of Edinburgh, 51 Little France Crescent, Edinburgh, EH16 4SA, UK

## Abstract

**Background:**

Oesophageal cancer is a major clinical problem with a generally poor prognosis. As a result there has been interest in combining surgery with neoadjuvant chemotherapy to try and improve outcomes, although the current evidence for benefit is inconsistent. We aimed to compare, in a non-randomised study, the post-operative complication rate and short and long-term survival of patients who underwent surgical resection for carcinoma of the oesophagus and types I and II carcinoma of the oesophago-gastric junction with or without neo-adjuvant chemotherapy.

**Methods:**

Details of all resections for oesophageal/junctional (types I and II) adenocarcinoma or squamous cell carcinoma between April 2000 and July 2006 were collected prospectively. Data from patients with T3 and/or N1 disease who underwent either neoadjuvant chemotherapy (NAC) or not (non-NAC) were compared. Data were analysed using Kaplan-Meier plots, Mann-Whitney U-test, Cox Regression modelling, and Chi-squared test with Yates' correction where sample sizes <10.

**Results:**

167 patients were included (89 NAC and 78 non-NAC). The in-hospital post-operative mortality rate of the NAC group (n = 2 deaths; 2.2%) was significantly lower (p = 0.045) than the non-NAC group (n = 6 deaths; 7.7%). Most deaths were due to cardio-respiratory complications; however, there was no significant difference in rates of chest infections, anastomotic leaks, wound infections, re-operations, readmission to ITU or overall complications between the two groups. Although both the two-year survival rate (60.7%) and long-term survival of NAC patients (median survival = 793 days; 95% CI = 390–1196) was greater than non-NAC patients (two-year survival rate = 48.7%; median survival = 554 days; 95% CI = 246–862 respectively), these differences were not statistically significant.

**Conclusion:**

This non-randomised study demonstrated that NAC was associated with a significant reduction in post-operative inpatient mortality rate. Whether this can be explained by a decreased co-morbidity in NAC patients or a protective phenomenon associated with NAC remains unclear. This study also demonstrated a greater two-year survival rate and overall median survival time following NAC but this was not statistically significant.

## Background

Surgery remains the treatment of choice for potentially curable oesophageal cancer, although long term survival rates are still poor [1-4]. As a result there has been increasing interest in combining surgery with neo-adjuvant chemotherapy (NAC) [1,4-6]. The potential benefits of NAC administration include improvement of swallowing, resulting in better patient nutrition, and down-staging of the primary tumour and elimination of micro-metastases, thus increasing the likelihood of a curative resection.

Randomised controlled trials and meta-analyses investigating the effect of NAC on oesophageal adenocarcinoma (ACC) and squamous cell carcinoma (SCC) have been inconsistent [1,4-6]. The MRC OEO2 trial demonstrated an increase in the two-year survival rate and median survival duration of patients who received two pre-operative cycles of cisplatin and 5-fluorouracil (5FU) [4]. However, another large trial failed to demonstrate a survival advantage with a similar regimen [5]. In the UK, current guidelines suggest that pre-operative chemotherapy for ACC or SCC of the oesophagus and oesophago-gastric junction (OGJ) (types I and II) should be considered [7].

In April 2000, we began a policy of treating selected patients in the South-East of Scotland with oesophageal or Type I/II OJG ACC/SCC with two cycles of pre-operative cisplatin and 5FU and we report here our results.

## Methods

### Patients

Details of all patients with oesophageal and OGJ cancer in the Lothian and Borders area are collected prospectively. Using this database, we analysed the demographics, post-operative complication rates and survival rates of those patients who underwent potentially curative resection of oesophageal or type I/II OGJ ACC or SCC between April 2000 and July 2006. During this time the indications for pre-operative chemotherapy were either T3 or N1 disease without evidence of distant metastases on pre-operative endoscopic ultrasound, CT or laparoscopic ultrasound scanning. We compared specifically the data of those patients with T3 and/or N1 disease who had received neo-adjuvant chemotherapy (NAC group) with those patients who did not receive neo-adjuvant chemotherapy either for personal reasons or medical contra-indications e.g. significant co-morbidity, in particular cardiac disease (non-NAC group). The chemotherapy regimen consisted of 2 pre-operative cycles of 5-FU and cisplatin, given 2 weeks apart. Surgery followed within 5 weeks. All patients with oesophageal carcinoma and type I carcinoma of the OGJ underwent an Ivor Lewis subtotal oesophagectomy with two field lymphadenectomy. Patients with type II OGJ carcinoma underwent either the same procedure or an oesophagogastrectomy through a left thoraco-abdominal incision, depending on the extent of tumour above and below the OGJ. This latter group had a level-two abdominal lymphadenectomy and local clearance of lymph nodes around the hiatus and up to the level of transection of the oesophagus. Survival was calculated in days from the date of endoscopic diagnosis. To investigate the possibility of tumour downstaging by neo-adjuvant chemotherapy, pre-operative staging was compared to final histology for each group. The Scottish Intercollegiate Guidelines on staging of oesophageal cancer were used [7].

### Statistics

The Statistical Package for Social Services Version 12 was used to analyse the data. Mann-Whitney U test was used to compare patient demographics between groups. Chi-squared test was used to determine any difference in the rate of post-operative complications between patient groups. Yates' correction for continuity was applied where sample size was less than 10. Univariate Kaplan-Meier analysis was used to assess patient survival in different patient groups and tumour stages. Any patient variable associated with a p value of < 0.1 on univariate Kaplan-Meier analysis was included in a multivariate Cox Regression model. Statistical significance was set at p < 0.05.

## Results

### Patient Demographics

The study included 167 patients, 89 of whom received NAC (NAC group) and 78 who did not (non-NAC group). There were no significant differences in patient sex, tumour stage (as determined post-operatively by a Consultant Pathologist) and histological tumour type between the two patient groups (Table 1). However, the NAC group were significantly younger than the non-NAC group (median age 60 yrs; range 30–79 yrs vs median age 65 yrs; range 37–82 yrs; p < 0.01) (Table 1).

**Table 1:** Demographics of the two patient groups. Age is expressed as medians with ranges in parentheses.

		**NAC (n = 89)**	**Non-NAC (n = 78)**	**p value**
**AGE (years)**		60 (30–79)	65 (37–82)	P < 0.01
**SEX**	**Male**	70	69	0.09
	**Female**	19	9	
**STAGE**	**I**	3	3	0.92
	**IIA**	20	16	
	**IIB**	6	6	
	**III**	50	44	
	**IV**	10	9	
**TYPE**	**Adenocarcinoma**	70	19	0.23
	**Squamous Cell Carcinoma**	67	11	

### Tumour Downstaging

In 75.3% of the NAC patients and 70.5% of the non-NAC patients, the pre-operative T stage matched the final post-operative histopathological T stage (p = 0.49). Similarly, 68.5% of the NAC patients and 59.0% of the non-NAC patients had matching pre- and post-operative N stages (p = 0.199). Thus, 27.0% of NAC patients and 17.9% of the non-NAC patients had either been over-staged pre-operatively or, in the case of the NAC patients, had undergone tumour 'downstaging. (p = 0.17) (Table 2).

**Table 2:** The number and percentage of patients whose T stage, N stage or overall stage was predicted pre-operatively to be higher than the stage determined post-operatively by histopathological examination.

	**NAC group (n = 89)**	**Non-NAC group (n = 78)**	**p value**
**Pre-op T > Post-op T**	12 (13.5%)	15 (19.2%)	0.31
**Pre-op N > Post-op N**	20 (22.5%)	12 (15.4%)	0.25
**Overall Pre-op stage > Overall Post-op stage**	24 (27.0%)	14 (17.9%)	0.17

### Post-Operative Complications

**Figure 1:**
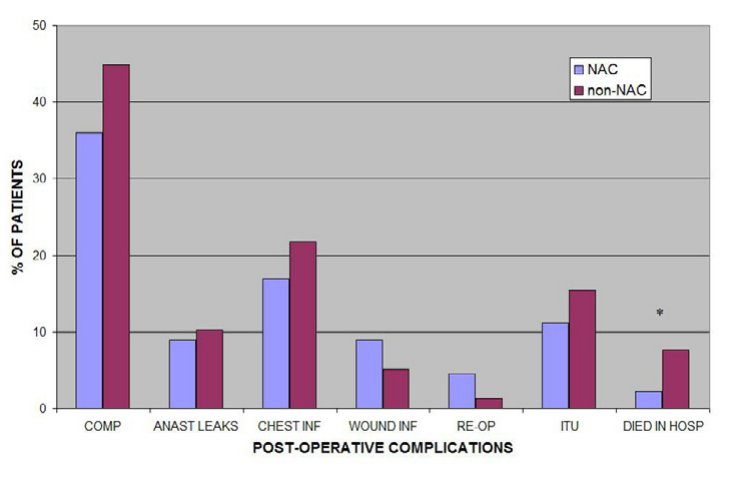
**Post-operative complication rates in the two patient groups**. (COMPS = overall post-operative complications: ANAST LEAKS = anastamotic leaks; CHEST INF = chest infections = WOUND INF = wound infections; RE-OP = patients who required a 're-operation'; ITU = number of patients re-admitted to ITU; DIED IN HOSP = number of patients who died in hospital; * = p = 0.045).

There was no significant difference in the incidence of anastomotic leaks (NAC: n = 8, 9.0%; non-NAC: n = 8, 10.3%, p = 0.59), wound infections (NAC: n = 8, 9.0%; non-NAC: n = 4; 5.1%; p = 0.51), chest infections (NAC: n = 15, 16.9%; non-NAC: n = 17, 21.8%; p = 0.42), re-admissions to ITU (NAC: n = 10, 11.2%; non-NAC: n = 12, 15.4%; p = 0.31), re-operations (NAC: n = 4, 4.5%; non-NAC: n = 1, 1.3%; p = 0.31) or overall post-operative complications (NAC: n = 32, 36.0%; non-NAC: n = 35, 44.9%; p = 0.24) between the two patient groups (Fig. 1). However, the NAC group did demonstrate a significantly lower post-operative in-hospital mortality rate (n = 2, 2.2%) compared with the non-NAC group (n = 6, 7.7%; p = 0.045). Of the 8 patients who died in hospital, 4 died due to pneumonia (all from the non-NAC group), a further 3 died due to cardio-respiratory arrest following a past history of ischaemic heart disease (2 non-NAC group and 1 NAC group), and 1 patient died following a significant upper gastrointestinal haemorrhage (NAC group).

### Patient Survival

**Figure 2:**
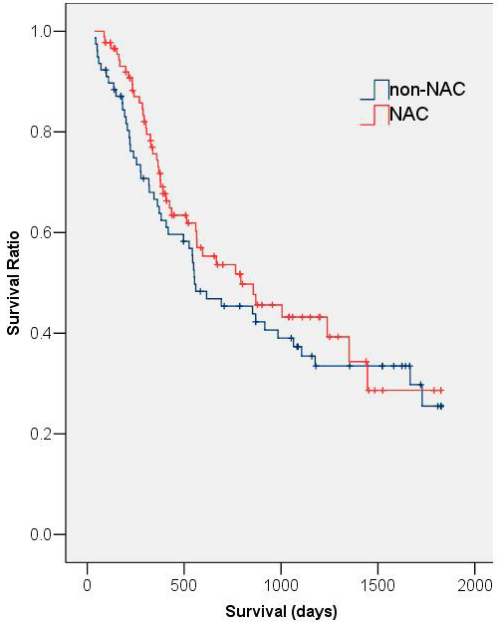
Kaplan-Meier survival curves of NAC and non-NAC groups (p = 0.37).

**Figure 3:**
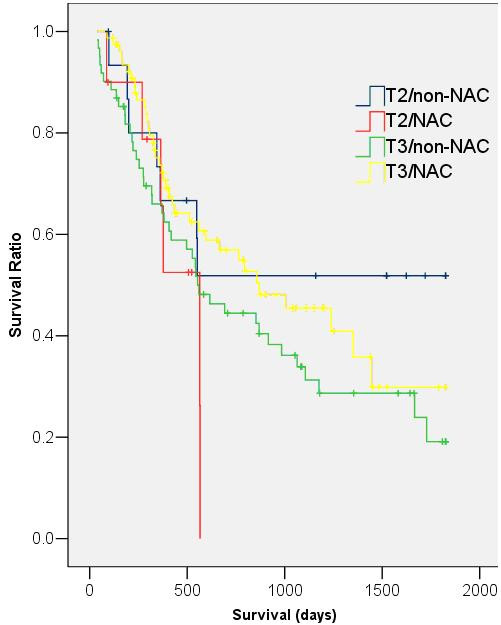
**Kaplan-Meier survival curves of NAC and non-NAC patients with T2 and T3 disease**. (T2/non-NAC: median survival: 552 days, 95% CI = 409–695 days; T2/NAC: median survival: 565 days, 95% CI = 361–769 days; T3/non-NAC: median survival: 554 days, 95% CI = 356–752 days; T3/NAC: median survival: 870 days, 95% CI = 407–1333 days; p = 0.65).

**Figure 4:**
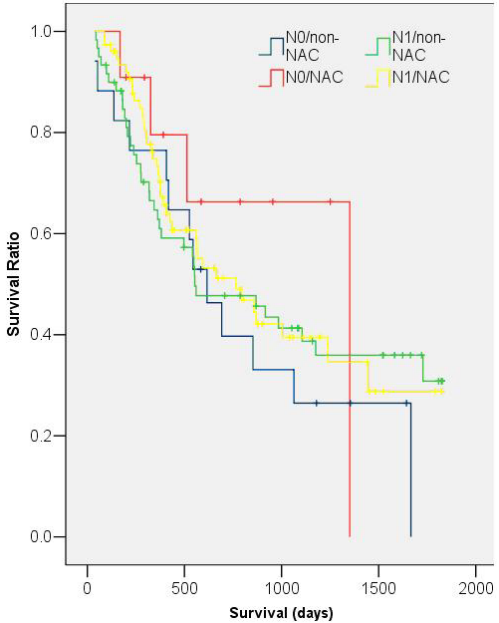
**Kaplan-Meier survival curves of NAC and non-NAC patients with N0 and N1 disease**. (N0/non-NAC: median survival: 616 days, 95% CI = 407–825; N0/NAC: median survival: 1351 days, 95% CI = 1351 days; N1/non-NAC: median survival: 554 days, 95% CI = 136–971 days; N1/NAC: median survival: 765 days, 95% CI = 417–1113 days; p = 0.16)

Median survival of the NAC group was 793 days (95% CI = 390–1196 days) compared with 554 days (95% CI = 246–862 days) in the non-NAC group. However, this difference did not reach statistical significance (p = 0.37) (Fig. 2). Two year survival rates for the NAC and non-NAC groups were 60.7% and 48.7% respectively (p = 0.16). This trend for improved survival in the NAC group was consistent when data were grouped and analysed by individual T and N stages (Figs. 3 &4). Univariate analysis demonstrated that patient age, sex, histological tumour type and pre-operative tumour stage did not significantly affect patient survival. Only post-operative stage as determined histopathologically affected patient survival significantly (p < 0.01). A Cox Regression model including post-operative stage and NAC status was generated, but this did not demonstrate a significant effect of NAC on the odds ratio (OR) of patient death (OR = 0.84; 95% CI = 0.55 – 1.27; p = 0.40).

## Discussion

Although this non-randomised study of patients undergoing oesophago-gastrectomy demonstrated that the overall complication rate in those patients not receiving NAC was greater than those who received NAC, these differences were not statistically significant and the overall rates are similar to those observed in other studies [4-6]. Our study did, however, demonstrate a significantly lower in-hospital mortality rate in the NAC group compared with the non-NAC group. The higher post-operative mortality of the non-NAC group may be attributed to a greater number of chest infections that, although not statistically significant, may have resulted in more systemic compromise in the non-NAC group, which in turn may have had a higher co-morbidity. This hypothesis is supported by the observation that the NAC group was significantly younger than the non-NAC group (median age 60 vs 65 yrs). Another possible explanation is that NAC is associated with a protective pre-conditioning phenomenon, which subsequently improves patients' post-operative recovery. A similar theory has been suggested in transplantation, where recent studies have demonstrated that by up-regulating the stress protein heme-oxygenase-1, using toxins such as curcumin, inflammatory injury and cellular apoptosis post-transplant are reduced [8,9].

Importantly, NAC administration was associated with increases in both two-year survival rate and overall median survival duration, although these increases were not statistically significant. These trends were also seen when the effect of NAC on specific stages of disease was examined. Relatively small patient numbers and a lack of statistical power may simply explain the lack of statistical significance. However, it is interesting to note that the difference in median survival duration and 2-year survival rate between the two treatment groups was greater than that observed in the MRC OEO2 trial (239 vs 107 days and 12% vs 9% respectively), a trial which demonstrated statistical significance [4]. The operative mortality reported in our study is also lower than that reported in the MRC OEO2 trial (5% vs 10% respectively). Explanations for the better figures we report may include management within a multi-disciplinary team, standardised staging, agreed treatment protocols and the utilisation of an experienced surgical team with appropriate ITU support.

Our results also suggested that NAC may 'downstage' tumours although this finding was also not statistically significant. This phenomenon of 'downstaging' may also be an explanation of the improved survival.

## Conclusion

In conclusion, our study demonstrates the successful regional implementation of NAC in the treatment of oesophageal cancer, and suggests a trend towards improved survival following the use of NAC. We have shown the ability to translate the results of clinical trials into routine practice and also illustrated the importance of audits following the introduction of new treatment regimens. Larger patient numbers may be required to show conclusively a statistically significant improvement in median survival duration. However, although the difference in median survival following NAC administration is not *statistically *significant in this study, for each individual patient it is likely to be *clinically *significant. Finally, the lower post-operative mortality associated with neoadjuvant chemotherapy is an interesting observation that warrants further basic science investigation.

## Competing interests

The author(s) declare that they have no competing interests.

## Authors' contributions

BPH and RJES performed data analysis. SPB, GWC and ACdB were the surgeons involved in patient care. LW and HAP were the oncologists involved in patient care. All authors were involved in the collection of data and in preparation of the manuscript.
